# The Lucky Ones: A Report of Two Cases of Intraorbital Foreign Bodies

**DOI:** 10.7759/cureus.16685

**Published:** 2021-07-28

**Authors:** Niki Wai Wye Ho, Mae-lynn Catherine Bastion, Mushawiahti Mustapha, Othmaliza Othman

**Affiliations:** 1 Department of Ophthalmology, Hospital Universiti Kebangsaan Malaysia, Kuala Lumpur, MYS

**Keywords:** foreign bodies, orbit, accidents, ocular trauma, ocular injury

## Abstract

Intraorbital foreign bodies are a common complication of eye trauma. If improperly managed, it may lead to severe complications. In this case series, the first case is an intraconal foreign body after being hit by a stone, and the second case is an intraconal foreign body from a metallic piece of a crane wheel. We discuss the role of imaging in confirming the presence of the foreign body, localizing it, and guiding when surgery is indicated. Our two cases showed differences in the management approach, as the first case had multiple issues requiring multiple procedures. However, the second case had a relatively specific pathology, management, and outcome. With adequate treatment and a bit of luck, the visual outcome can be satisfactory.

## Introduction

An intraorbital foreign body refers to a foreign body occurring within the orbit, but outside the globe. Anatomically, it is divided into two compartments by the extraocular muscles: intraconal and extraconal [1]. This entity usually occurs after high-velocity injuries, such as industrial accidents, but can rarely occur following trivial trauma. A retained foreign body can lead to severe complications, the worst being a loss of the eye function. In general, inorganic matter such as metal and glass are well tolerated, and if not causing any effects, may be left in situ. In contrast, organic matter elicits intense inflammation, needing urgent removal [2]. We discuss two cases of intraorbital foreign body, their management, and three months’ postoperative visual outcome.

## Case presentation

Case 1 

**Figure 1:**
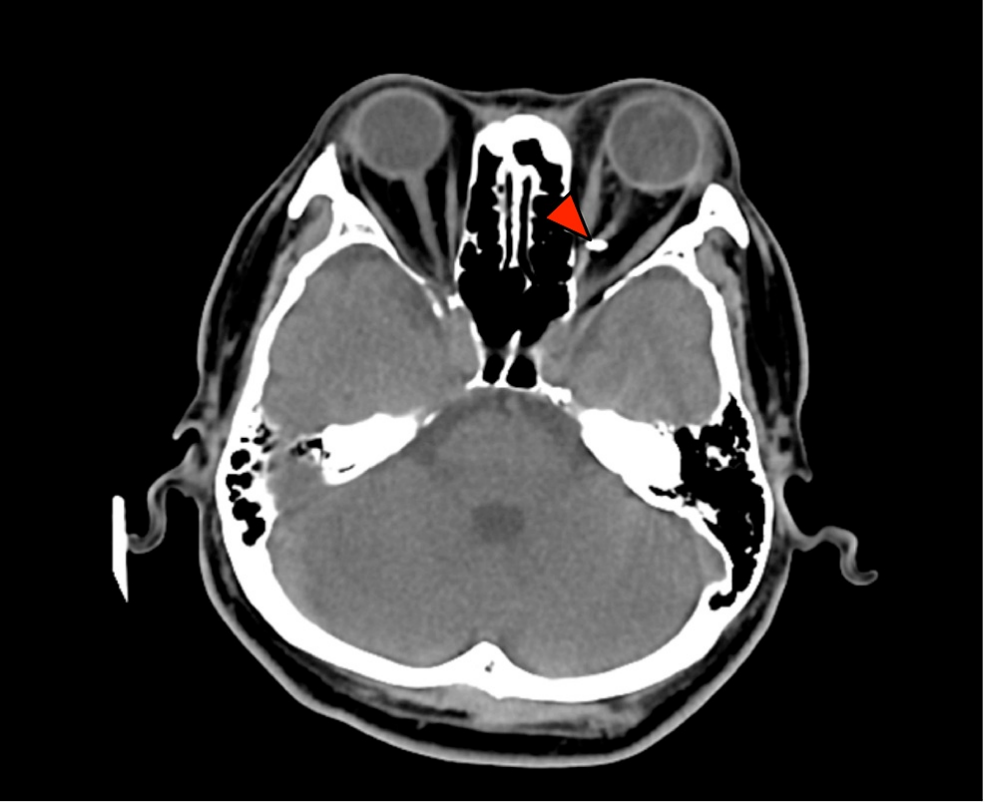
CT scan demonstrating a foreign body (arrow)

A 52-year-old male had an injury to his left eye after being hit by a stone while cutting grass. His left vision upon presentation was hand movement with a relative afferent pupillary defect. The anterior segment was normal. Funduscopy showed a vitreous haemorrhage with a poor view of the fundus. A computed tomography (CT) scan revealed a small intraconal foreign body, in proximity to the optic nerve (Figure 1).

Case 1 Management

A pars planar vitrectomy for vitreous haemorrhage clearance was performed. However, he had a persistent postoperative vitreous haemorrhage and subsequently developed high intraocular pressure secondary to ghost cell glaucoma with a cataract. He underwent an anterior chamber washout, vitreous haemorrhage clearance, endolaser, endocyclophotocoagulation, and cataract surgery. Postoperatively, visual acuity improved to 6/12 and intraocular pressure was controlled with anti-glaucoma eyedrops. At two months post-trauma, when intraocular pressure was controlled, orbitotomy was done and revealed a small stone located posteriorly close to the optic nerve, near the orbital apex, but removal was aborted. Postoperatively, visual acuity was 6/18. After five months, he developed a rhegmatogenous retinal detachment and a successive pars planar vitrectomy with intraocular tamponade was performed.

Case 1 Outcome

Postoperatively at three months, visual acuity was 6/18 with a flat retina. His latest best-corrected visual acuity 18 months post-trauma was 6/18. 

Case 2

**Figure 2:**
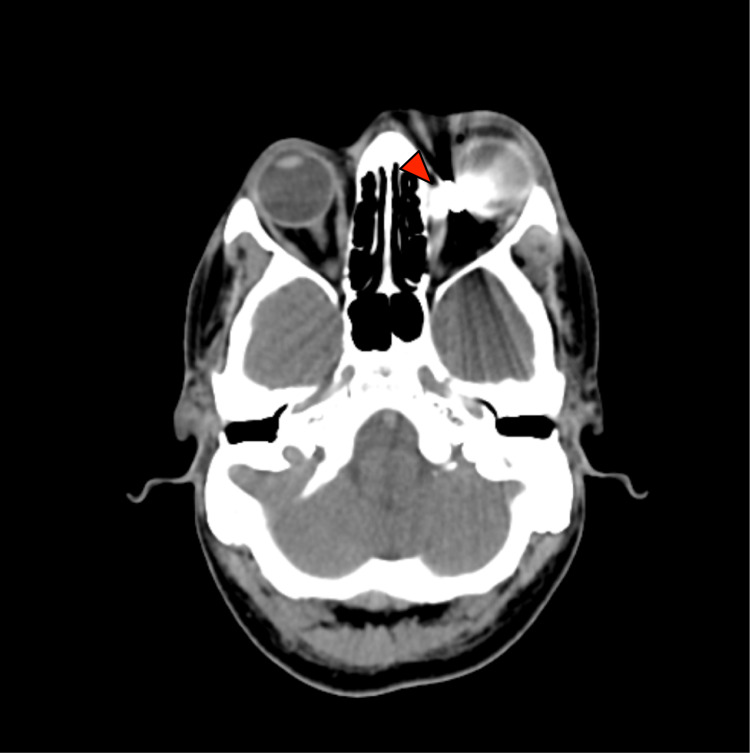
CT imaging demonstrating a foreign body (arrow)

A 27-year-old male had an injury in his left eye by a metallic piece from a crane wheel. His left vision upon presentation was hand movement with no relative afferent pupillary defect. He had a self-sealed scleral laceration, retinal hole, vitreous haemorrhage, and commotio retinae. CT scanning showed a metallic foreign body located in the intraconal region near the medial orbital wall (Figure 2).

Case 2 Management

Laser retinopexy for a retinal hole was done. The vitreous haemorrhage and commotio retinae were conservatively managed. The foreign body, being metallic, was found to be embedded in the Tenon space and was removed. 

Case 2 Outcome

Postoperatively at three months, visual acuity was 6/6. 

## Discussion

Penetrating lid or orbital injuries should always raise suspicion of an intraorbital foreign body, mainly if the trauma was of high velocity. The patient may either be symptomatic or asymptomatic. 

It is well known that the severity of injury in penetrating trauma of the orbit is often underestimated in physical examination [3]. Therefore, radiological imaging plays an important role. Plain radiography is helpful to confirm the presence of the foreign body. CT scanning helps to pinpoint the exact location (Figures 1, 2), hence providing a guide to access during surgery. Magnetic resonance imaging (MRI) is contraindicated in metallic foreign bodies as it may pose a risk of migration and injury to orbital structures.

Surgery is planned based on several factors. These include the nature of the foreign body, which, if organic, warrants surgery; the location of the foreign body, if posteriorly located and inorganic, should be left alone; and presence of other foreign body-related complications such as intense inflammatory reactions, abscess, chronic discharging sinus, and optic nerve compression [4].

Early diagnosis, management, and extraction of the foreign body greatly influence the outcome. However, it is crucial to weigh the benefits versus risks. Posteriorly located foreign bodies tend to confer a worse prognosis because of associated traumatic optic neuropathy [5].

Both cases were discussed as they showed similarities but also differences, especially in the management approach. In the first case, the foreign body was small, inorganic in nature, located posteriorly with no evidence of foreign body-related complications. There was no clear evidence of optic nerve compression as the foreign body was tiny with no intraorbital bony fragments, and the patient had a good vision after the vitreous haemorrhage clearance. He also had multiple ocular issues, such as secondary glaucoma with uncontrolled intraocular pressure initially, which delayed the orbitotomy procedure. During orbitotomy, removal of the foreign body was aborted, as there were signs of optic nerve compromise during the attempts. However, as it was deemed not harmful, thus it was left in situ to avoid the risk of iatrogenic optic nerve injury. Our management was similar to Fulcher et al.’s case series demonstrating six conservatively managed patients because of posteriorly located inorganic foreign bodies [6]. Siedlecki et al. also demonstrated a similar case of a metallic foreign body in the left orbital apex abutting the optic nerve, who presented asymptomatically 30 years post-trauma with a good vision of 6/7.5 and was conservatively managed [7]. In contrast, the foreign body in the second case was anteriorly located; thus, removal was relatively straightforward, without much risk.

In these two cases, we would also like to highlight the contrasting severity of complications in penetrating trauma to the orbit. Despite trauma with a small foreign body, in some cases, the patient might develop multiple sequelae or issues and may even require multiple procedures, as per the first case. However, in the second, they may have a relatively specific pathology, management, and outcome. Nonetheless, the clinicians’ adequate judgment and treatment are exceptionally crucial, affecting the outcome.

With adequate treatment, whether conservative or surgical, intraorbital foreign bodies may have an improvement in visual acuity, with 74% having a vision of 6/36 or better [4]. In our two cases, both had a vision of 6/18 or better at three months postoperatively, despite both having hand movement vision at presentation.

## Conclusions

Good history, examination, and imaging are essential in diagnosing intraorbital foreign bodies. Not all intraorbital foreign bodies warrant surgical removal, and weighing the benefits and risks pre-operatively is essential. With proper management and a bit of luck, the visual outcome is satisfactory.
